# *Kodamaea ohmeri* as an Emerging Human Pathogen: A Review and Update

**DOI:** 10.3389/fmicb.2021.736582

**Published:** 2021-09-10

**Authors:** Menglan Zhou, Yanbing Li, Timothy Kudinha, Yingchun Xu, Zhengyin Liu

**Affiliations:** ^1^Department of Clinical Laboratory, State Key Laboratory of Complex Severe and Rare Diseases, Peking Union Medical College Hospital, Chinese Academy of Medical Sciences, Peking Union Medical College, Beijing, China; ^2^Beijing Key Laboratory for Mechanisms Research and Precision Diagnosis of Invasive Fungal Diseases, Beijing, China; ^3^School of Biomedical Sciences, Charles Sturt University, Orange, NSW, Australia; ^4^NSW Health Pathology, Regional and Rural, Orange Base Hospital, Orange, NSW, Australia; ^5^Department of Infectious Disease, Peking Union Medical College Hospital, Chinese Academy of Medical Sciences, Peking Union Medical College, Beijing, China

**Keywords:** *Kodamaea ohmeri*, infection, epidemiology, microbiology, treatment

## Abstract

**Background:***Kodamaea ohmeri*, previously known as *Pichia ohmeri* or *Yamadazyma ohmeri*, has been regarded as an emerging human pathogen in recent decades, and has caused various types of infections with high mortality. This study systematically reviewed all the published cases of *K. ohmeri* infection, aiming to have a better understanding of the clinical and epidemiological characteristics of the organism.

**Methods:** All the published literature (as of March 31, 2021) on *K. ohmeri*, in four databases: PubMed, Embase, Web of Science, and CNKI, were systematically reviewed to select appropriate studies for summarizing the demographic information, clinical and microbiological characteristics of relevant infections.

**Results:** A total of 51 studies involving 67 patients were included for final analysis, including 49 sporadic cases and two clusters of outbreaks. Neonates and the elderly constituted the majority of patients, and fungemia was the dominant infection type. Comorbidities (like malignancy, diabetes, and rheumatism), invasive operations, previous antibiotic use and prematurity, were commonly described in patients. Gene sequencing and broth microdilution method, were the most reliable way for the identification and antifungal susceptibility testing of *K. ohmeri*, respectively. Amphotericin B and fluconazole were the commonest antifungal therapies administered. The calculated mortality rates for *K. ohmeri* infection was higher than that of common candidemia.

**Conclusion:** In this study, we systematically reviewed the epidemiology, clinical characteristics, microbiological features, treatment, and outcomes, of all the published cases on *K. ohmeri*. Early recognition and increased awareness of *K. ohmeri* as an emerging human pathogen by clinicians and microbiologists is important for effective management of this organism.

## Introduction

*Kodamaea ohmeri*, which belongs to *Saccharomycetes* family, is also formerly known as *Pichia ohmeri* or *Yamadazyma ohmeri*. It is usually isolated from the environment, and is commonly used in the food care industry for fermentation. It is generally believed that *K. ohmeri* was first isolated from a patient’s blood in 1998 (Bergman et al., 1998), and some decades later, it has become an emerging human pathogen that can cause life-threatening infections, especially in immunocompromised patients. Sporadic cases of human infections by this organism have been reported worldwide, including fungemia, endocarditis, catheter-related bloodstream infection, and cutaneous infection, among several others (Kanno et al., 2017; Ni et al., 2018; Yu et al., 2020). Moreover, nosocomial outbreaks of *K. ohmeri* in the pediatric intensive care unit (ICU) have been reported (Liu et al., 2012; Chakrabarti et al., 2014). Invasive infections caused by this organism have been reported with significant mortality as high as 50% (Otag et al., 2005; Lee et al., 2007). Despite its increasing role as a human pathogen in the clinical setting, the clinical and epidemiological characteristics of *K. ohmeri* infection are not well understood. Furthermore, the identification of *K. ohmeri* has presented some challenges in Microbiology laboratories, specifically in that the different identification methods previously used by most clinical labs were time-consuming or had low accuracy levels (Chakrabarti et al., 2014; Zhou et al., 2019). Early recognition of the organism and administration of appropriate treatment are important considerations in the management of this rare fungal infection.

Herein, we systematically reviewed all the published cases on *K. ohmeri* infections in humans, aiming to have a better overview of the epidemiology, clinical characteristics, microbiological features, treatment, and outcomes of these cases. We hope to provide empirical treatment recommendations based on the detailed analysis of the current data, and make an early call to clinicians and microbiologists for the recognition of *K. ohmeri* as an emerging human pathogen.

## Methods

### Search Strategy

To obtain published studies related to *K. ohmeri* infections (as of March 31, 2021), we searched through PubMed, Embase, Web of Science, and CNKI databases, using the following terms: (*Kodamaea* OR *Pichia* OR *Yamadazyma*) AND *ohmeri*.

### Study Selection

Two independent reviewers (Zhou M and Li Y) performed a systematic literature review of potentially relevant studies on *K. ohmeri*. Studies were screened by title and abstract, and those that met the following criteria were included for further analysis: (a) published in English or Chinese language, (b) confirmed *K. ohmeri* infection in humans, and (c) provision of data on patients’ clinical characteristics, microbiology features, treatment, and outcomes. Exclusion criteria included studies with one or more of the following conditions: (a) published in languages other than English or Chinese, (b) organism not isolated from humans, (c) epidemiology or surveillance studies, (d) colonization but not infection, and finally (e) reviews and conference papers that didn’t provide full information of the infection.

### Data Extraction and Statistical Analysis

Data from eligible studies were extracted by the two independent reviewers. Microsoft Excel v.2019 (Microsoft Corp., United States) was used for data entry and analysis. The extracted data included the study type, year of publication, author, country and district, patient demographic information (age and gender), clinical characteristics (underlying diseases and conditions, hospital department, previous antibiotic use, treatment strategy, and outcome), and infection and microbiology data (infection site, identification methods, antifungal susceptibility test (AST) results, inflammatory indicators, and other pathogens isolated at the same time). Statistical analysis was performed with the χ^2^ test.

## Results

### Systemic Literature Search

**FIGURE 1:**
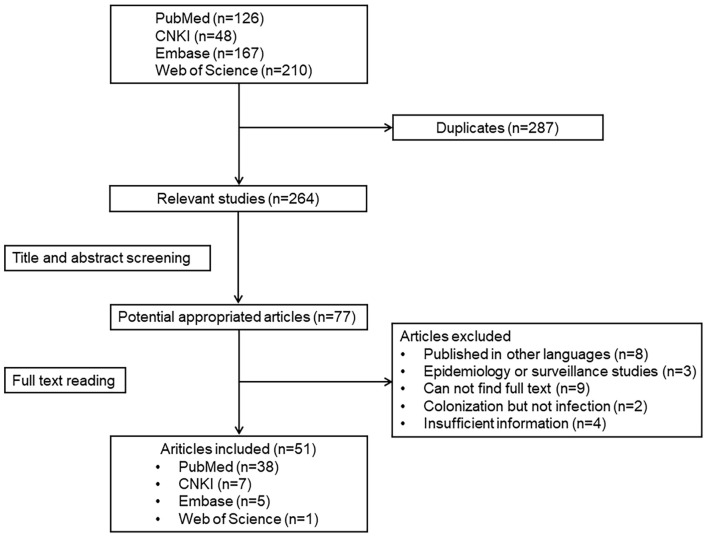
Results of systemic literature search.

A total of 551 relevant articles on *K. ohmeri* were identified in the four databases (PubMed, Embase, Web of Science, and CNKI), with period of publication ranging from January 1975 to March 2021. After exclusion of duplicates and title/abstract screening, 77 articles were selected for full-text reading. Among these, eight were published in languages other than English or Chinese, nine were without available full texts, three focused on epidemiology or surveillance, two involved colonization rather than infection, and four provided insufficient information. Consequently, 51 studies were selected for further analysis (Figure 1), including 49 sporadic cases (Jin and Jin, 1994; Bergman et al., 1998; Choy et al., 2000; Matute et al., 2000; Hitomi et al., 2002; Huang, 2002; Joao et al., 2002; Liu et al., 2002; Puerto et al., 2002; Reina et al., 2002; Shin et al., 2003; Han et al., 2004; Ostronoff et al., 2006; Taj-Aldeen et al., 2006; Lee et al., 2007; Mahfouz et al., 2008; De Barros et al., 2009; Poojary and Sapre, 2009; Yang et al., 2009; Chiu et al., 2010; Menon et al., 2010; Shaaban et al., 2010; Shang et al., 2010; Yanghua et al., 2010; Al-Sweih et al., 2011; Gonzalez-Avila et al., 2011; Sundaram et al., 2011; Zhang et al., 2011; Santino et al., 2013; Xiao et al., 2013; Biswal et al., 2015; Bokhary and Hussain, 2015; Capoor et al., 2015; Das et al., 2015; Distasi et al., 2015; Cao et al., 2016; Giacobino et al., 2016; Ma et al., 2016; Vivas et al., 2016; Fernandez-Ruiz et al., 2017; Kanno et al., 2017; Huang et al., 2018; Ni et al., 2018; Tashiro et al., 2018; Diallo et al., 2019; Hou, 2019; Saud Al-Abbas et al., 2019; Al-Salameh et al., 2020; Yu et al., 2020) and two clusters of infection (Otag et al., 2005; Liu et al., 2012).

### Distribution of Included Studies

**FIGURE 2:**
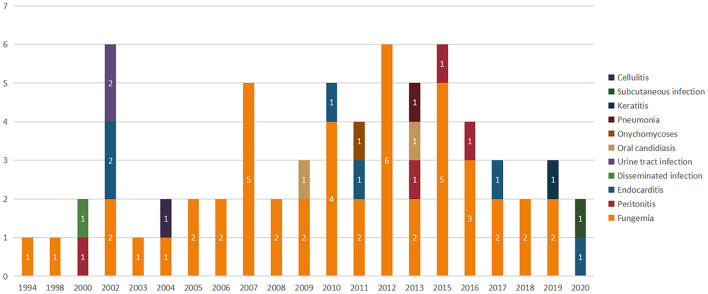
Timeline of published *K. ohmeri* infections. No., number of publications.

The 51 studies included for final analysis involved 67 patients in total. Eight (15.7%), 11 (21.6%), and 32 (62.7%) of the studies were reported from Europe, America, and Asia, respectively. Among the publications in Asia, half (16/32, 50.0%) was reported from China, followed by India (6/32, 18.8%). All the published cases, based on timeline and infection types, are displayed in Figure 2. Based on the four databases, in chronological order, the first *K. ohmeri* fungemia was reported in 1994 in China (article in Chinese) (Jin and Jin, 1994) rather than in the United States in 1998 as generally believed (Bergman et al., 1998). Since 2002, there has been at least one *K. ohmeri* infection case reported every year. However, whenever a nosocomial outbreak occurred, the number of infections would increase significantly, such as in year 2012. A total of 49 studies reported sporadic cases or case series of various infections, and two reported on potential *K. ohmeri* outbreak in the neonatal and pediatric intensive care unit (NICU and PICU), in China and Turkey, respectively. The specific clinical characteristics of all the *K. ohmeri* infections included in this review are detailed in Supplementary Table 1.

### Epidemiology of the *K. ohmeri* Infection Cases

The *K. ohmeri* cases were reported worldwide, with the majority being from Asia (46/67, 68.5%), especially in east and southeast Asia countries (24 in China, three in Japan, six in Korea, seven in India, two in Turkey, and one each in Kuwait, Lebanon, Malaysia, and Kingdom of Saudi Arabia). Although the first *K. ohmeri* fungemia case in PubMed was reported in the United States in 1998, only 12 cases have been reported in the Americas since then. The remaining nine cases were reported in Europe and none was reported from Africa. Among the 67 infection cases, the majority (62.7%; 42/67) of patients were male. The age of infected patients ranged from neonates to 84 years old. Among all the sporadic cases, the elderly (older than 65 years old) made up a larger proportion (16/59, 27.1%) compared to the other age groups. However, the two clusters of outbreaks occurred in neonates and children (Otag et al., 2005; Liu et al., 2012). In 38 patients, admission department information was available for analysis. Among these, 14 patients were admitted in the ICU and 9 patients in the emergency department. Five patients were initially admitted to the hematology department due to their hematologic malignancies. Two patients each, were admitted to the respiratory, cardiovascular surgery and oncology departments. One patient each, was reported from the department of dermatology, ophthalmology, pediatric, and vascular surgery (Table 1).

**TABLE 1:** Epidemiological characteristics of *K. ohmeri* infection cases.

Classification	Distribution	No.	%
District	Asia	46	68.5
	Americas	12	17.9
	Europe	9	13.4
	Total	67	100
Gender			
	Male	42	62.7
	Female	25	37.3
	Total	67	100
Department			
	ICU	14	36.8
	Emergency	9	23.7
	Hematology	5	13.2
	Respiratory	2	5.3
	Cardiovascular surgery	2	5.3
	Oncology	2	5.3
	Pediatric	1	2.6
	Dermatology	1	2.6
	Ophthalmology	1	2.6
	Vascular surgery	1	2.6
	Total	38	100

*No., number.*

### Clinical Characteristics of *K. ohmeri* Infections

*Kodamaea ohmeri* caused both invasive and non-invasive infections, with invasive infections accounting for the majority (62/67, 92.5%) of the cases reported. And among the invasive infections, fungemia (46/62, 74.2%) dominated (Jin and Jin, 1994; Bergman et al., 1998; Hitomi et al., 2002; Huang, 2002; Shin et al., 2003; Han et al., 2004; Otag et al., 2005; Ostronoff et al., 2006; Taj-Aldeen et al., 2006; Lee et al., 2007; Mahfouz et al., 2008; De Barros et al., 2009; Poojary and Sapre, 2009; Yang et al., 2009; Chiu et al., 2010; Shaaban et al., 2010; Shang et al., 2010; Al-Sweih et al., 2011; Zhang et al., 2011; Liu et al., 2012; Xiao et al., 2013; Biswal et al., 2015; Capoor et al., 2015; Das et al., 2015; Distasi et al., 2015; Ma et al., 2016; Vivas et al., 2016; Fernandez-Ruiz et al., 2017; Kanno et al., 2017; Huang et al., 2018; Tashiro et al., 2018; Diallo et al., 2019; Hou, 2019), followed by endocarditis (7/62, 11.3%) (Joao et al., 2002; Reina et al., 2002; Yanghua et al., 2010; Sundaram et al., 2011; Cao et al., 2016; Ni et al., 2018; Al-Salameh et al., 2020), and peritonitis (4/62, 6.4%) (Choy et al., 2000; Xiao et al., 2013; Bokhary and Hussain, 2015; Giacobino et al., 2016). Other infection types were less frequent, including urinary tract infection (*n* = 2) (Liu et al., 2002; Puerto et al., 2002), pneumonia (*n* = 1) (Santino et al., 2013), keratitis (*n* = 1) (Saud Al-Abbas et al., 2019), and one case of disseminated infection (Matute et al., 2000). Fever and chills were the most common clinical features in patients who developed invasive infections. In fungemia cases, respiratory distress and disturbance of consciousness were reported as the patient progressed to sepsis. However, a fungemia case in which the patient never presented with pyrexia was also reported (Distasi et al., 2015). Besides, different infection types had different local symptoms, i.e., abdominal pain and hematuria often occurred in patients who had peritonitis and urinary tract infection. If the infection site was explicit (such as the catheter insertion site and wound infection), local redness, swelling, and pain often occurred (Supplementary Table 1).

The duration of hospitalization ranged from 4 days to 3 months, with an average of 27.8 days. For patients who recovered (65.0%, 28 out of 43 with available data), the average hospitalization duration was 29.1 days, while for patients who died of infection (15/43, 34.9%), the average was 25.9 days. White blood cell (WBC) count was a common inflammatory indicator for infection, and around half of the cases reported this data. In the 32 cases with available WBC count data, 25.0% (8/32) was within the normal range and 65.6% (21/32) of the patients had leukocytosis, among which 61.9% (13/21) of the increase did not exceed 50% of the upper limit of the reference range (15 × 10^9^/L). On the other hand, three patients presented with leukopenia, and two of them had hematological malignancies (Supplementary Table 1).

The two reported clusters of outbreaks included two and six fungemia cases, in children and pre-mature infants, respectively (Otag et al., 2005; Liu et al., 2012). All these eight patients presented with fever, accompanied by hemostasis, difficulty in breathing, and other respiratory distress syndrome. Four neonates had significant anemia, progressive decrease in platelets, and increased enzymatic indexes in liver function tests (Liu et al., 2012). The average length of hospital stay in the two clusters of outbreaks was 28 and 21 days. In the outbreak involving six neonates, all the patients recovered after caspofungin and fluconazole treatment (Liu et al., 2012), while in the other cluster outbreak, the 8-month-old male infant failed to respond to fluconazole and died on the 21st day of hospitalization (Otag et al., 2005).

As for the non-invasive infections, two cases of oral mucositis (Menon et al., 2010; Santino et al., 2013) and one each of onychomycoses (Gonzalez-Avila et al., 2011), cellulitis (Han et al., 2004) and subcutaneous infection (Yu et al., 2020), were reported. Despite being a non-invasive infection, a patient who developed cellulitis due to multiple infections involving *K. ohmeri*, *Staphylococcus aureus*, *Proteus mirabilis*, and *Enterococcus*, developed tissue necrosis and a purulent discharge, and finally died of the infection (Han et al., 2004). However, due to the limited number of cases, the mortality rate of *K. ohmeri* non-invasive infections remain largely unclear (Supplementary Table 1).

### Underlying Diseases and Potential Risk Factors

Most cases were reported in immunocompromised patients who had malignancy (including leukemia, lymphoma, and other solid tumors), rheumatoid disease, diabetes, chronic viral infections (such as HBV, HCV, and HIV), or other serious infectious diseases (such as meningitis, pneumonia, and bacterial sepsis) and organ dysfunction syndrome (the most common are renal and hepatic insufficiency). Patients with these underlying diseases often required immunosuppressive therapy which thus impaired the immune system function. Among the cases reported, infectious disease (19/67, 28.4%) is the most common, followed by malignancy (17/67, 25.4%), and diabetes mellitus (10/67, 14.9%). Rheumatoid disease (4/67, 6.0%) and organ dysfunction (4/67, 6.0%) also accounted for a considerable proportion. It should also be noted that *K. ohmeri* infection has been reported in immunocompetent individuals (Diallo et al., 2019), albeit much less frequently (Supplementary Table 1).

In addition to underlying diseases, some patients with *K. ohmeri* infections had also undergone various invasive procedures during hospitalization. Specifically, all kinds of implants (central venous catheter, peripheral catheter, pacemaker, bioprosthetic mitral valve, urethral catheter, and implanted organs), were potential risk factors for *K. ohmeri* infections. Among all the cases, 46.3% (31/67) of the patients had been implanted with at least one kind of catheter, and removal of the catheters or other implants contributed to a better prognosis. Therefore, catheter removal was the first-line therapeutic strategy for *K. ohmeri* catheter-related infections. Furthermore, 22.4% (15/67) of the patients who had received tracheotomy or mechanical ventilation were under immunosuppressive therapy, receiving chemotherapeutic drugs and steroids. As for the peritonitis cases, all the patients had received peritoneal dialysis. Prematurity was also an important risk factor for neonates, with 90.9% (10/11) of neonates developing sporadic *K. ohmeri* infections, having been born prematurely. This was also observed in one of the outbreak clusters, among which all six neonates were preterm (Liu et al., 2012; Supplementary Table 1).

### Isolation and Identification

Since most *K. ohmeri* infections presented as fungemia, blood was the most common isolation source (51/67, 76.1%, followed by catheter tip culture which was done in 20.9% (14/67) of the cases. The third most common isolation source was peritoneal fluid (4/67, 6.0%), which was described in all the four peritonitis patients. *K. ohmeri* was also isolated from nail or skin culture in three cases, including one each of phlebitis, subcutaneous infection, and onychomycoses. In two cases each, the organism was isolated from wound tissue, oral swabs, respiratory secretions (including sputum and bronchoalveolar fluid), and urine. It is worth mentioning that in a neonatal fungemia case, *K. ohmeri* was also isolated from the mother’s high vaginal swab, indicating a possible infection route (Biswal et al., 2015).

The conventional culture-based method, using CHROMagar coloration was performed in 43.3% (29/67) of the cases. Biochemical methods such as VITEK 2 compact (42/67, 62.7%) and API 20C (31/67, 46.3%), were the most commonly used commercial methods. ATB ID 32C and matrix-assisted laser desorption/ionization time of flight mass spectrometry (MALDI-TOF MS), were both used in 6.0% (4/67) of the cases. Gene sequencing was regarded as the gold standard for *K. ohmeri* identification, and was performed in more than half of the cases reviewed here (40/67, 59.6%). The internal transcribed spacer (ITS) region (ITS1 and/or ITS2 genes) was most frequently used in 47.5% (19/40) of the cases, followed by 5.8S rDNA (12/40, 30.0%), D1/D2 region of 28S rDNA (11/40, 27.5%), and 18S rDNA (8/40, 20.0%). In 27.5% (11/40) of the cases, more than one gene was sequenced to accurately identify the species. In two cases, the sequenced gene was not specified. In the majority of cases (52/67, 77.6%), a combination of two or more identification methods were used simultaneously, which reduced the misidentification rate compared to using one only.

### Antifungal Susceptibility Test

Antifungal susceptibility test was performed in 67.2% (45/67) of the cases. The recommended broth microdilution method was used in 44.4% (20/45) of the cases, followed by E-test (6/45, 13.3%), commercial methods such as ATB fungus 3 (7/45, 15.6%), Sensititre Yeast One (3/45, 6.6%), VITEK compact system (2/45, 4.4%), disk diffusion (1/45, 2.2%), and EIKEN examination kit (1/45, 2.2%). There were five cases in which the antifungal susceptibility method used was not specified.

Since there is no established breakpoint for *K. ohmeri* by the European Committee on Antimicrobial Susceptibility Testing (EUCAST) or Clinical and Laboratory Standards Institute (CLSI), we excluded four cases which only provided susceptibility category as either susceptible or resistant without providing minimum inhibitory concentration (MIC) values when analyzing AST results. The MICs of fluconazole as obtained from 40 patients, ranged from 1 to >128 mg/L, with MIC_50_ of 8 mg/L and MIC_90_ of 32 mg/L. On the other hand, MICs of voriconazole determined in isolates from 26 patients ranged from 0.015 to 2 mg/L, with MIC_50_ of 0.06 mg/L and MIC_90_ of 0.5 mg/L. MICs of itraconazole (from 30 patients) ranged from 0.008 to <2 mg/L, with MIC_50_ of 0.125 mg/L and MIC_90_ of 0.5 mg/L, while that of posaconazole (*n* = 5 patients) ranged from 0.012 to 0.06 mg/L, with MIC_50_ of 0.03 mg/L and MIC_90_ of 0.06 mg/L. For micafungin (*n* = 11 patients), the MIC ranged from 0.03 to 1 mg/L, with MIC_50_ of 0.06 mg/L and MIC_90_ of 0.125 mg/L. In the case of caspofungin (*n* = 15 patients), the MIC ranged from 0.125 to ≥16 mg/L, with MIC_50_ of 0.25 mg/L and MIC_90_ of 4 mg/L, which was much higher than that of anidulafungin (*n* = 5 patients) which ranged from 0.06 to 1 mg/L, with MIC_50_ of 0.125 mg/L and MIC_90_ of 1 mg/L. Amphotericin B (*n* = 39 patients) MICs ranged from 0.008 to 1 mg/L, with MIC_50_ of 0.25 mg/L and MIC_90_ of 0.5 mg/L, compared to <0.02 to 4 mg/L for 5-flucytosine (*n* = 21 patients), with MIC_50_ of 0.5 mg/L and MIC_90_ of 2 mg/L. MICs of miconazole and ketoconazole were only tested on 1 patient each, and were 0.5 mg/L and 0.06 mg/L, respectively (Table 2). The specific AST results with available MIC data are detailed in Supplementary Table 2.

**TABLE 2:** Antifungal susceptibility profiles of *K. ohmeri* isolates.

Antifungals	MIC range (mg/L)	MIC_50_ (mg/L)	MIC_90_ (mg/L)	No.
Fluconazole	1– >128	8	32	40
Voriconazole	0.015–2	0.06	0.5	26
Itraconazole	0.008– <2	0.125	0.5	30
Posaconazole	0.012–0.06	0.03	0.06	5
Micafungin	0.03–1	0.06	0.125	11
Caspofungin	0.125– ≥16	0.25	4	15
Anidulafungin	0.06–1	0.125	1	5
Amphotericin B	0.008–1	0.25	0.5	39
5-flucytosine	<0.02–4	0.5	2	21
Ketoconazole	0.06	–	–	1
Miconazole	0.5	–	–	1

*No., number of isolates.*

### Treatment and Outcome

For patients who had catheter implantation, catheter removal as a treatment strategy was undertaken in 61.3% (19/31) of the patients. As for cutaneous infection and endocarditis, surgical excision of the granuloma or vegetation was performed. Amphotericin B was the most frequently used first line antifungal treatment, being administered in 44.6% (29 out of 65 cases with available data) of the patients, followed by fluconazole (23/65, 35.4%), caspofungin (9/65, 13.8%), voriconazole (5/65, 7.7%), micafungin (3/65, 4.6%), and itraconazole (3/69, 4.3%). Anidulafungin, ketoconazole, and isavuconazole were only reported in the treatment of one patient each (1.5%). Combined antifungal drug treatment was applied in 24.6% (16/65) of cases. Amphotericin B and fluconazole were the commonest therapy combination, and was used in 13.8% of the patients (9/65). Fluconazole combined with echinocandins was used in three patients (4.6%), while two patients (3.1%) received a combination of multiple azole drugs. Only one patient each (1.5%) received a combination of micafungin and amphotericin B, and amphotericin B and voriconazole.

The majority (52/67, 77.6%) of the patients in this review had received antibiotic therapy previously. In invasive infection cases, this rate rose to 82.2% (51/62). Some of the antibiotics were administered empirically, whilst others were used for treating complicated bacteremia. Vancomycin (12/52, 23.1%) was the most commonly used antibiotic among these cases, followed by piperacillin (8/52, 15.4%), and meropenem (8/52, 15.4%). As for cephalosporins, ceftazidime was used in five patients, ceftriaxone in four patients, cefazolin in three patients, and cefotaxime in two patients.

Based on available data, the overall mortality of *K. ohmeri* infection is 30.8% (20 out of 65 patients), among which invasive infection accounted for 95.0% (19/20) of the deaths. Among these invasive cases, fungemia accounted for the majority (14/19, 73.7%) of the deaths, followed by endocarditis (*n* = 2), peritonitis (*n* = 2), and systematic disseminated infection (*n* = 1).

## Discussion

In the last two decades, infections caused by rare fungi have increased significantly (Miceli et al., 2011; Pande et al., 2017). *K. ohmeri*, belonging to the *Saccharomycetes* family, is an ascosporogenous yeast and a teleomorph of *Candida guilliermondii var. membranaefaciens*. Among the *Kodamaea* species (including *K. anthrophila*, *K. kakaduensis*, *K. laetipori*, *K. nitidulidarum*, and *K. ohmeri* five species), *K. ohmeri* is the only one that can grow under 37°C and infect humans. In recent years, there has been an increase in the number of *K. ohmeri* infections, with high mortality rates, and various invasive infections have been reported worldwide. Therefore, this emerging human pathogen has aroused widespread concern in the field of microbiology and infection, and a systematic review summarizing the clinical and microbiological characteristics of some sporadic *K. ohmeri* infections in humans was published last year (Ioannou and Papakitsou, 2020). However, inclusion of information on nosocomial outbreaks due to *K. ohmeri* deserves attention especially in a hospital setting, hence this review. As previously mentioned, two outbreaks of *K. ohmeri* infections have been reported, including one in China (Liu et al., 2012) and another in Turkey (Otag et al., 2005), both of which involved neonates and children. Moreover, an epidemiological study of a large cluster of fungaemia cases due to *K. ohmeri* in a pediatric department has also been published, in which as many as 38 neonates were infected. Considering that *K. ohmeri* is an emerging important human pathogen, we systematically reviewed all published *K. ohmeri* infection cases through four databases, including 67 patients in total, and summarized the clinical and microbiological characteristics of all the cases, hoping to provide a more comprehensive and detailed update of this rare organism.

Fungal infection can hasten the death of patients, and like other non-candida yeasts, *K. ohmeri* can cause life-threatening infections mainly in immunocompromised individuals. The calculated mortality rate of *K. ohmeri* infection was 30.8% (20/65) for all cases (30.6% for invasive infection), which is higher than common candidemia (21.2%) (Yamamoto et al., 2013). Therefore, *K. ohmeri* infection should be considered a critical condition in hospitalized patients and those with chronic diseases. Comorbidities (like malignancy, diabetes, and rheumatism) and central venous catheters (CVC) implantation, are the commonest predisposing factors. Invasive procedures which can break the skin mucosal barrier, including surgery, catheterization, and dialysis, can also be a potential risk factor for *K. ohmeri* infection. Treatment of *K. ohmeri* infection includes removal of the risk factors (such as CVC and mechanical ventilation) and administration of appropriate antifungal agents. Removal of the CVC proved to be a highly effective measure in certain cases, with catheter-related fungemia clearance after catheter removal (without antifungal therapy) (Lee et al., 2007).

Various antifungal regimens were used in the treatment of *K. ohmeri* infection. Since there were no breakpoints for *K. ohmeri*, we compared the MIC values with the breakpoints of *Candida* species. The susceptibility of the organism to fluconazole varied between studies, while amphotericin B often exhibited a low MIC value (Park et al., 2008). However, strains isolated from an outbreak in India showed a contrary result, in which 86.8% (33/38) of the isolates had a relatively higher MIC of amphotericin B (1 mg/L), possibly due to the widespread use of this antifungal drug in India because of the low cost (Chakrabarti et al., 2014). Echinocandins also exhibited good *in vitro* activity according to the limited data available. In the nosocomial neonatal infection in China (Liu et al., 2012), five of the six patients were treated with caspofungin, and all recovered. However, some cases in which patients were treated with *in vitro* susceptible antifungal drugs (fluconazole and amphotericin B) which failed to eliminate the fungemia, have been described (Distasi et al., 2015). However, it should be noted that *in vitro* antifungal susceptibility for a drug does not necessarily translate into effectiveness the *in vivo* setting, as other parameters such as the infection site and the patient’s tolerance, affect the effectiveness of the drug. Furthermore, the selection of different AST methods can affect the MIC values significantly. According to previous surveillance studies, the standard broth microdilution method and some commercial methods can lead to differences in MIC values (Tashiro et al., 2018; Zhou et al., 2019). Therefore, as for rare fungi like *K. ohmeri*, the standard broth microdilution method may be more reliable and accurate. The clinical antifungal treatment strategy should be adjusted promptly according to the susceptibility report rather than empirical drug use.

As a rare pathogen isolated in the clinical setting, the identification of *K. ohmeri* was problematic in the early days. *K. ohmeri* was most commonly mistaken for *Candida albicans*, *Candida glabrata*, and *Candida tropicalis*, based on the colony morphology. In most clinical microbiology labs, the CHROMagar *Candida* chromogenic growth medium is a useful tool for identifying *Candida* species based on the different colored colonies. *K. ohmeri* can grow yeast-like colonies on CHROMagar, the color of which change from pink/lilac to blue in 2–3 days, which can be distinguished from *Candida glabrata* (lilac) and *Candida tropicalis* (blue). However, this color change takes time and often needs a continuous observation. Therefore, the misidentification rate of CHROMagar in identifying *K. ohmeri* can be pretty high (up to 100%) if only one single observation is performed in routine work (Zhou et al., 2019). Biochemical methods such as API 20C, VITEK 2 compact, and ATB ID32C, were widely used in the identification of this organism in several microbiology labs. Although most of these methods are very reliable in identifying common *Candida* species, misidentification of *K. ohmeri* as *C. glabrata*, *C. lusitaniae*, *C. albicans*, *C. guilliermondi*, etc., has been reported (Lee et al., 2007; Chakrabarti et al., 2014; Zhou et al., 2019). The development of MALDI-TOF MS has enabled rapid identification of *Candida* species in clinical laboratories, and it has been successfully used for identifying *K. ohmeri* in several cases (Distasi et al., 2015; Kanno et al., 2017; Huang et al., 2018; Hou, 2019). A previous study has evaluated the two mainstream MALDI-TOF MS instruments in identifying *K. ohmeri*, and found that both Vitek MS and Bruker system with the protein extraction method for sample preparation, can be used as a fast and accurate tool for *K. ohmeri* identification with an accuracy >96% (Zhou et al., 2019). Nevertheless, gene-based molecular method is still the gold standard in identifying rare species, as the ESCMID/ECMM joint clinical guideline suggests (Arendrup et al., 2014). The D1/D2 regions, ITS regions were used for precise identification of *K. ohmeri* in most of the studies, when an unreliable result was obtained initially (Lee et al., 2007).

To conclude, we systematically reviewed all the published cases on *K. ohmeri* infections. By providing a detailed overview of the epidemiology, clinical and microbiological characteristics of *K. ohmeri* infections, we hope to raise the awareness level of clinicians and microbiologists on *K. ohmeri* infections due to its rareness, high mortality and different resistance pattern from usual yeasts.

Our study has some limitations. Firstly, we excluded epidemiology and surveillance studies due to insufficient data. Thus, the current study mainly included sporadic case reports for analysis. Secondly, the number of cases reviewed is quite small, especially for non-invasive infections, which affects the statistical power of the findings.

## Author Contributions

MZ and YL wrote the manuscript. TK, YX, and ZL revised the manuscript. All authors approved the final version of the manuscript.

## Conflict of Interest

The authors declare that the research was conducted in the absence of any commercial or financial relationships that could be construed as a potential conflict of interest.

## Publisher’s Note

All claims expressed in this article are solely those of the authors and do not necessarily represent those of their affiliated organizations, or those of the publisher, the editors and the reviewers. Any product that may be evaluated in this article, or claim that may be made by its manufacturer, is not guaranteed or endorsed by the publisher.
