# Supplementary material for: Kodamaea ohmeri as an Emerging Human Pathogen: A Review and Update
Source: Front Microbiol. 2021 Sep 10;12:736582. doi: 10.3389/fmicb.2021.736582 (PMC8461310; doi:10.3389/fmicb.2021.736582)
Supplement: Supplementary file 1 [file Data_Sheet_1.docx]

Table S1. Clinical characteristics of *Kodamaea ohmeri* infection of all included cases.

|  |  |  |  |  |  |  |  |  |  |  |  |
| --- | --- | --- | --- | --- | --- | --- | --- | --- | --- | --- | --- |
| **No.** | **Year** | **District** | **Age(Y)** | **Gender** | **Underlying disease** | **Potential risk factors** | **Infection types** | **Isolation sources** | **Treatments** | **Outcome** | **Ref No.** |
| **sporadic** |  |  |  |  |  |  |  |  |  |  |  |
| 1 | 2020 | China | 55 | F | rheumatoid arthritis | use of methylprednisolone | subcutaneous infection | skin | surgery | recovered | (4) |
| 2 | 2020 | USA | 35 | M | prolonged QT syndrome, chronic hepatitis C | intravenous drug use | endocarditis | blood | isavuconazole | recovered | (48) |
| 3 | 2019 | France | 81 | F | cognitive disorder | NM | fungemia | bronchoalveolar fluid, blood | VRZ | recovered | (10) |
| 4 | 2019 | Malaysia | 57 | F | DM | NM | keratitis | corneal scraping sample | FCZ, AMB | recovered | (11) |
| 5 | 2019 | China | 25 | M | hypoxic-ischemic encephalopathy, pneumonia, multiple organ dysfunction | CVC, mechanical ventilation | fungemia | CVC tip | catheter removed, CAP | recovered | (49) |
| 6 | 2018 | Japan | 60s | F | severe extended burn | autograft | fungemia | wound lesion, blood | AMB, MICA | died | (12) |
| 7 | 2018 | China | 63 | M | gastric cancer | NM | fungemia | blood | FCZ, anidulafungin | died | (50) |
| 8 | 2017 | Japan | 58 | F | rheumatoid arthritis, breast cancer, pancreatitis | anti-rheumatic medication, mechanical ventilation, CVC | fungemia | blood | catheter removed, MICA | recovered | (2) |
| 9 | 2017 | China | 57 | M | hemophagocytic lymphohistiocytosis | use of methylprednisolone | endocarditis | blood | VRZ | recovered | (3) |
| 10 | 2017 | Spain | 63 | M | acute leukemia, COPD | CVC, parenteral nutrition | fungemia | CVC tip | catheter removed, FCZ, AMB | died | (14) |
| 11 | 2016 | Colombia | 54 days | M | acute necrotizing enterocolitis | prematurity, mechanical ventilation | fungemia | blood | catheter removed, CAP | recovered | (13) |
| 12 | 2016 | Brazil | 71 | F | DM | peritoneal dialysis | peritonitis | peritoneal fluid | NM | died | (15) |
| 13 | 2016 | China | 69 | F | asthma, rheumatic heart disease | use of glucocorticoid | endocarditis | blood | VRZ | died | (16) |
| 14 | 2016 | China | 11 | M | Wilson's disease | use of steroids | fungemia | blood | FCZ, CAP | recovered | (42) |
| 15 | 2015 | India | 32 | M | AML | chemotherapy | fungemia | blood | AMB, MICA | died | (17) |
| 16 | 2015 | Italy | 80 | M | AMI, colon carcinoma | mechanical ventilation, CVC | fungemia | blood | FCZ, AMB | died | (18) |
| 17 | 2015 | India | 75 | M | NM | surgery, CVC | fungemia | blood | AMB | recovered | (19) |
| 18 | 2015 | KSA | 9 | M | steroid-resistant nephrotic syndrome | use of cyclosporine A | peritonitis | peritoneal fluid | catheter removed, CAP | recovered | (47) |
| 19 | 2015 | India | 50 | M | large B cell lymphoblastic lymphoma, DM | chemotherapy | fungemia | blood | AMB | recovered | (53) |
|  |  |  | 2 days | F | NM | mechanical ventilation | fungemia | blood, mother’s high vaginal swab | AMB | recovered |  |
| 20 | 2013 | Italy | 77 | M | pneumonia | use of steroids | pneumonia | sputum | VRZ, FCZ | recovered | (20) |
|  |  |  | 78 | F | lung cancer | chemotherapy | oral mucositis | oral mucosal lesion | FCZ, ICZ | recovered |  |
| 21 | 2013 | China | 61 | F | uremia | peritoneal dialysis | peritonitis | peritoneal fluid | catheter removed, ICZ | died | (51) |
|  |  |  | 46 | M | hepatitis B | mechanical ventilation | fungemia | blood | catheter removed, CAP | recovered |  |
|  |  |  | 62 | M | gastric perforation, acute renal insufficiency | surgery, mechanical ventilation | fungemia | blood | VRZ, AMB | died |  |
| 22 | 2011 | Mexico | 51 | F | DM | NM | onychomycoses | nail specimen | NM | NM | (22) |
| 23 | 2011 | India | 40 days | M | neonatal seizures, enterocolitis | prematurity | endocarditis | blood | AMB | died | (21) |
| 24 | 2011 | Kuwait | 13 days | F | NM | prematurity, mechanical ventilation | fungemia | blood | AMB | recovered | (23) |
| 25 | 2011 | China | 75 | F | DM | CVC, mechanical ventilation | fungemia | blood, catheter tip | catheter removed, FCZ, CAP | recovered | (43) |
| 26 | 2010 | China | 71 | M | DM, coronary artery disease, Cushing’s syndrome | peripheral catheter | fungemia | blood | FCZ, AMB | recovered | (24) |
|  |  |  | 58 | F | esophageal squamous cell carcinoma, pneumonia | surgery, CVC | fungemia | blood | FCZ | died |  |
| 27 | 2010 | USA | 34 | M | asthma, tracheo-esophageal fistula | peripheral catheter | fungemia | blood | catheter removed, MICA | recovered | (25) |
| 28 | 2010 | China | 55 | M | alcoholic hepatitis, peptic ulcer | surgery, CVC | fungemia | blood | catheter removed, CAP | recovered | (26) |
| 29 | 2010 | China | 43 | M | rheumatoid heart disease, hepatitis B | NM | endocarditis | blood | ICZ | recovered | (52) |
| 30 | 2009 | Brazil | 3 | F | purulent peritonitis, pneumonia | surgery, CVC | fungemia | blood, catheter tip | catheter removed, AMB | recovered | (27) |
| 31 | 2009 | India | 38 | F | HIV | NM | oral candidiasis | oral swab | FCZ | NM | (29) |
| 32 | 2009 | Netherland | 71 | M | DM | NM | fungemia | blood | AMB | recovered | (30) |
| 33 | 2008 | India | neonate | M | patent ductus arteriosus | prematurity, mechanical ventilation, umbilical arterial and venous catheters | fungemia | blood, catheter tip | catheter removed, ventilator removed, FCZ, AMB | died | (28) |
| 34 | 2008 | Lebanon | 38 | F | AML, haemochromatosis | chemotherapy, poly-site catheter | fungemia | blood | catheter removed, AMB | recovered | (31) |
| 35 | 2007 | Korea | 11 | M | Burkitt’s lymphoma | chemotherapy, CVC | fungemia | blood, catheter tip | FCZ | died | (8) |
|  |  |  | 41 | M | alcoholic ketoacidosis, tuberculosis | CVC | fungemia | blood | catheter removed | recovered |  |
|  |  |  | 47 | M | pneumonia, DM, chronic renal failure | CVC | fungemia | blood, catheter tip | AMB, FCZ. | died |  |
|  |  |  | 4 | F | tetralogy of Fallot | CVC, immunotherapy | fungemia | blood | AMB, FCZ | died |  |
|  |  |  | 0 | F | NM | prematurity, umbilical artery and vein catheter | fungemia | blood | catheter removed | recovered |  |
| 36 | 2006 | Brazil | 58 | F | CML | chemotherapy, CVC | fungemia | blood, catheter tip | catheter removed, AMB | recovered | (32) |
| 37 | 2006 | USA | neonate | F | NM | prematurity, mechanical ventilation | fungemia | blood | AMB | recovered | (33) |
| 38 | 2004 | USA | 14 | M | ALL | chemotherapy, CVC | fungemia | blood | catheter removed, FCZ | recovered | (34) |
|  |  |  | 74 | M | malignant fibrous histiocytoma | NM | cellulitis | wound | FCZ | died |  |
| 39 | 2003 | Korea | 59 | M | meningitis, pneumonia | surgery | fungemia | blood, swab from the skin | AMB | recovered | (35) |
| 40 | 2002 | Portugal | 42 | M | hepatitis C, peripheral vasculopathy | intravenous drug use, multiple skin lesions | endocarditis | blood, embolus | AMB | recovered | (36) |
| 41 | 2002 | Spain | 73 | M | non-Hodgkin’s lymphoma | indwelling catheter | urinary tract infection | urine | FCZ | recovered | (37) |
| 42 | 2002 | Japan | 84 | M | maxillary sinus squamous cell carcinoma | CVC | fungemia | blood, catheter tip | catheter removed, AMB | died | (38) |
| 43 | 2002 | USA | 76 | M | endocarditis | bioprosthetic mitral valve | endocarditis | blood | FCZ, AMB | recovered | (39) |
| 44 | 2002 | China | 76 | M | pneumonia | CVC | fungemia | blood, catheter tip | AMB | recovered | (44) |
| 45 | 2002 | China | 71 | M | DM | urethral catheter | urine tract infection | urine | FCZ | recovered | (45) |
| 46 | 2000 | Netherland | 71 | M | endocarditis | pacemaker | disseminated infection | blood, catheter tip | AMB | died | (40) |
| 47 | 2000 | China | 64 | M | NM | peritoneal dialysis | peritonitis | peritoneal fluid | catheter removed, FCZ, AMB | recovered | (41) |
| 48 | 1998 | USA | 48 | F | DM, post-renal transplantation | use of prednisone, CVC | fungemia | blood | AMB | died | (1) |
| 49 | 1994 | China | 64 | M | carcinoma of cecum | NM | fungemia | blood | KET | recovered | (46) |
| **cluster** |  |  |  |  |  |  |  |  |  |  |  |
| 1 | 2012 | China | neonate | M (n=3) F (n=3) | sepsis, meningitis | prematurity (n=6), ventilator support (n=4), PICC (n=5) | fungemia | blood (n=6) PICC tip (n=3) | CAP (n=5) FCZ (n=1) | recovered (n=6) | (6) |
| 2 | 2005 | Turkey | 8 months | M | encephalitis | NM | fungemia | blood | FCZ | died | (7) |
|  |  |  | 10 | M | B-cell ALL | CVC | fungemia | blood | AMB | recovered |  |
|  |  |  |  |  |  |  |  |  |  |  |  |

Y, years old; F, female; M, male; DM, diabetes mellitus; ALL, acute lymphoblastic leukemia; CML, chronic myelogenous leukemia; AML, acute myelogenous leukemia; AMI, actue myocardial infarction; COPD, chronic obstructive pulmonary disease; CVC, central venous catheter; PICC, peripherally inserted central venous catheter; FCZ, fluconazole; AMB, amphotericin B; ICZ, itraconazole; VRZ, voriconazole; CAP, caspofungin; MICA, micafungin; KET, ketoconazole; NM, not mentioned; The numbers in parentheses above indicate the number of patients.

Table S2. Summary of antifungal susceptibility results of *Kodamaea ohmeri* isolates.

|  |  |  |  |  |  |  |  |  |  |  |  |  |  |
| --- | --- | --- | --- | --- | --- | --- | --- | --- | --- | --- | --- | --- | --- |
| **No.** | **isolation No.** | **MIC values (mg/L)** | | | | | | | | | | | **Ref No.** |
|  |  | **FCZ** | **VRZ** | **ICZ** | **MCZ** | **KET** | **POS** | **MICA** | **CAP** | **ANI** | **AMB** | **5-FC** |  |
| 1 | 1-1 | 4 | 0.05 | ND | ND | ND | ND | ND | >2 | ND | 0.09 | ND | (10) |
| 2 | 2-1 | 8 | 0.06 | 0.25 | ND | ND | ND | 0.03 | 0.12 | ND | 1 | 0.5 | (12) |
| 3 | 3-1 | 4 | ND | 0.06 | 0.5 | ND | ND | 0.12 | ND | ND | 0.5 | ≤0.12 | (2) |
| 4 | 4-1 | 8 | ≤0.12 | ND | ND | ND | ND | ND | ND | ND | 0.5 | ND | (13) |
| 5 | 5-1 | 8 | 0.06 | ND | ND | ND | 0.02 | 0.12 | ND | 1 | 0.03 | ND | (14) |
| 6 | 6-1 | 32 | 0.12 | ND | ND | ND | ND | ND | 0.06 | ND | 0.5 | ND | (15) |
| 7 | 7-1 | 4 | 0.03 | 0.12 | ND | 0.06 | 0.06 | 1 | > 16 | 1 | 0.5 | < 0.03 | (18) |
| 8 | 8-1 | 4 | 0.015 | 0.25 | ND | ND | ND | ND | ND | ND | 0.12 | ND | (20) |
|  | 8-2 | >128 | 0.5 | 0.5 | ND | ND | ND | ND | ND | ND | 0.5 | ND |  |
| 9 | 9-1 | <16 | ND | <2 | ND | ND | ND | ND | ND | ND | <0.5 | <0.5 | (22) |
| 10 | 10-1 | 2 | <0.12 | ND | ND | ND | ND | ND | ND | ND | <0.25 | <1 | (21) |
| 11 | 11-1 | 4 | 0.047 | 0.12 | ND | ND | 0.012 | 0.12 | 0.25 | 0.064 | 0.023 | 0.032 | (23) |
| 12 | 12-1 | 64 | ND | 0.5 | ND | ND | ND | ND | ND | ND | 0.5 | <0.5 | (24) |
|  | 12-2 | 8 | 0.12 | 0.12 | ND | ND | ND | ND | ND | ND | 0.5 | <4 |  |
| 13 | 13-1 | 16 | ND | 0.25 | ND | ND | ND | ND | ND | ND | 0.25 | <0.5 | (25) |
| 14 | 14-1 | 4 | 0.12 | 0.12 | ND | ND | ND | ND | ND | ND | 0.5 | 4 | (26) |
| 15 | 15-1 | 8 | 0.12 | 0.12 | ND | ND | 0.063 | ND | 2 | 0.063 | 0.5 | ND | (27) |
| 16 | 16-1 | 4 | <0.063 | <0.12 | ND | ND | ND | ND | ND | ND | <0.5 | <4 | (28) |
| 17 | 17-1 | 16 | ND | 1 | ND | ND | ND | ND | ND | ND | ND | ND | (29) |
| 18 | 18-1 | 64 | ND | 0.5 | ND | ND | ND | ND | ND | ND | <0.5 | <0.5 | (30) |
| 19 | 19-1 | 32 | 0.5 | 0.5 | ND | ND | ND | 0.03 | 0.12 | ND | 0.25 | ND | (8) |
|  | 19-2 | 16 | 0.12 | 0.25 | ND | ND | ND | 0.06 | 0.25 | ND | 0.25 | ND |  |
|  | 19-3 | 4 | 0.03 | 0.12 | ND | ND | ND | 0.03 | 0.12 | ND | 0.25 | ND |  |
|  | 19-4 | 16 | 0.06 | 0.12 | ND | ND | ND | 0.03 | 0.12 | ND | 0.25 | ND |  |
|  | 19-5 | 2 | 0.03 | 0.12 | ND | ND | ND | 0.03 | 0.12 | ND | 0.5 | ND |  |
| 20 | 20-1 | 4 | ND | ND | ND | ND | ND | ND | ND | ND | 0.5 | ND | (32) |
| 21 | 21-1 | 32 | ND | 0.25 | ND | ND | ND | ND | ND | ND | 0.064 | <0.02 | (33) |
| 22 | 22-1 | 8 | ND | ND | ND | ND | ND | ND | ND | ND | 0.008 | ND | (7) |
|  | 22-2 | 8 | ND | ND | ND | ND | ND | ND | ND | ND | 0.008 | ND |  |
| 23 | 23-1 | 32 | ND | 0.25 | ND | ND | ND | ND | ND | ND | 0.5 | ND | (34) |
|  | 23-2 | 32 | ND | 0.008 | ND | ND | ND | ND | ND | ND | 1 | ND |  |
| 24 | 24-1 | 4 | ND | 0.5 | ND | ND | ND | ND | ND | ND | 0.5 | ND | (35) |
| 25 | 25-1 | 4 | ND | 0.03 | ND | ND | ND | ND | ND | ND | 0.25 | ≤0.12 | (39) |
| 26 | 26-1 | 4 | 0.03 | 0.06 | ND | ND | 0.03 | 0.12 | 0.5 | 0.12 | 0.5 | ≤0.06 | (47) |
| 27 | 27-1 | 64 | 2 | 0.5 | ND | ND | ND | ND | 0.12 | ND | <0.5 | ≤4 | (49) |
| 28 | 28-1 | 2 | 0.06 | 0.12 | ND | ND | ND | ND | ND | ND | 0.5 | <4 | (51) |
|  | 28-2 | 1 | 0.12 | 0.06 | ND | ND | ND | ND | ND | ND | 0.5 | <4 |  |
|  | 28-3 | 1 | 0.12 | 0.06 | ND | ND | ND | ND | ND | ND | 0.5 | <4 |  |
| 29 | 29-1 | 1 | 0.25 | ND | ND | ND | ND | ND | 0.25 | ND | 0.25 | 1 | (53) |
|  | 29-2 | 4 | 0.25 | ND | ND | ND | ND | ND | 0.25 | ND | 0.25 | 1 |  |
|  |  |  |  |  |  |  |  |  |  |  |  |  |  |

ND, no data; FCZ, fluconazole; AMB, amphotericin B; ICZ, itraconazole; VRZ, voriconazole; CAP, caspofungin; MICA, micafungin; KET, ketoconazole；MCZ, miconazole; POS, Posaconazole; ANI, anidulafungin; 5-FC, 5-flucytosine.
